# Aquaporin Protein-Protein Interactions

**DOI:** 10.3390/ijms18112255

**Published:** 2017-10-27

**Authors:** Jennifer Virginia Roche, Susanna Törnroth-Horsefield

**Affiliations:** Department of Biochemistry and Structural Biology, Center for Molecular Protein Science, Lund University, Box 124, 221 00 Lund, Sweden; jennifer.roche@biochemistry.lu.se

**Keywords:** aquaporin, protein-protein interactions, gating, trafficking, membrane channel, membrane protein

## Abstract

Aquaporins are tetrameric membrane-bound channels that facilitate transport of water and other small solutes across cell membranes. In eukaryotes, they are frequently regulated by gating or trafficking, allowing for the cell to control membrane permeability in a specific manner. Protein–protein interactions play crucial roles in both regulatory processes and also mediate alternative functions such as cell adhesion. In this review, we summarize recent knowledge about aquaporin protein–protein interactions; dividing the interactions into three types: (1) interactions between aquaporin tetramers; (2) interactions between aquaporin monomers within a tetramer (hetero-tetramerization); and (3) transient interactions with regulatory proteins. We particularly focus on the structural aspects of the interactions, discussing the small differences within a conserved overall fold that allow for aquaporins to be differentially regulated in an organism-, tissue- and trigger-specific manner. A deep knowledge about these differences is needed to fully understand aquaporin function and regulation in many physiological processes, and may enable design of compounds targeting specific aquaporins for treatment of human disease.

## 1. Introduction

Protein–protein interactions are fundamental for cellular function, playing key roles in virtually all biological processes, including gene transcription and translation, intracellular signaling, maintaining cellular organization, energy transduction and communication with the environment [1]. For aquaporins (AQPs), ubiquitous membrane-bound channels that facilitate the transmembrane diffusion of water and other small solutes, protein–protein interactions are crucial for their role in a number of human physiological processes, including urine concentration, maintaining eye lens transparency, and formation of tears, sweat and saliva. Traditionally, AQPs are divided into two families, the orthodox AQPs, permeable to water only and the Aquaglyceroporins that also conduct other small uncharged solutes, most notably glycerol. In addition, AQPs have been shown to conduct a variety of other molecules, for example gases (CO_2_ and NH_3_) [2,3,4], hydrogen peroxide [5], and metalloids such as arsenic, boron and silicon [4,6]. Interestingly, several studies have shown that under certain conditions, some AQPs can also function as gated ion channels [7,8,9,10], an activity that has been suggested to be involved in modulating production of cerebrospinal fluid [11].

**Figure 1:**
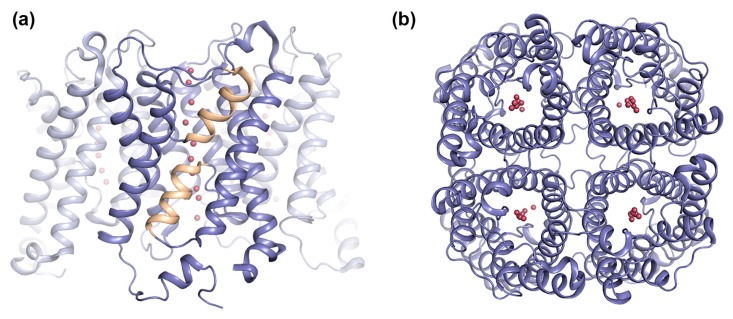
Overall structure of aquaporins (AQPs). Crystal structure of human AQP5 (PDB code 3D9S) showing the AQP tetramer viewed: (**a**) parallel to the membrane and (**b**) from the extracellular side. The AQP5 tetramer is shown in blue ribbon-representation with half-membrane spanning helices formed by loops B and E colored yellow. Water molecules in the pore are shown as red spheres.

Structural studies of prokaryotic as well as eukaryotic AQPs have revealed a highly conserved fold, consisting of six transmembrane helices surrounding a narrow substrate-conducting channel (Figure 1). In addition, two half-membrane spanning helices, formed by loops B and E dipping into the membrane from opposite sides, create a seventh pseudo-transmembrane segment. Through this channel, water and other uncharged solutes are selectively conducted in a single file along a concentration gradient, remarkably without permitting passage of protons. In the membrane, four AQP monomers assemble into a homo-tetramer, with each monomer functioning as an individual channel. The tetrameric assembly creates a fifth channel through the middle of tetramer. This central channel is of mostly hydrophobic nature and has been suggested to conduct gases, for example CO_2_ in plant AQPs [12].

**Figure 2:**
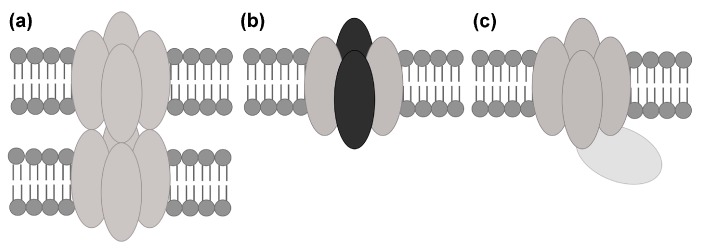
Types of AQP protein–protein interactions. AQP protein–protein interactions can typically be divided into: (**a**) interactions between AQP tetramers in supramolecular assemblies; (**b**) interactions between different AQP monomers (grey and black ovals) in hetero-tetramers and (**c**) transient interactions with regulatory proteins.

Despite this common structural architecture, eukaryotic AQPs have evolved to be differentially regulated depending on the organism and/or tissue they are expressed in. This includes regulation by conformational changes (i.e., gating) or by controlling the amount of AQPs present in a membrane through trafficking [12]. In some tissues, AQPs have also adopted other functions than facilitating transmembrane transport, for example the involvement of AQP0 in cell adhesion in the human eye lens [13]. Protein–protein interactions play crucial roles in AQP regulation as well as alternative AQP functions and can primarily be divided into three types: (1) interactions between AQP tetramers in supramolecular assemblies (junctions and arrays); (2) interactions between AQP monomers of different types, i.e., hetero-tetramerization; and (3) transient interactions with regulatory proteins (Figure 2). In this review, we will summarize recent knowledge concerning these three types of interactions and how they control AQP regulation and function. Focusing on the structure–function relationships of these interactions, we will discuss how differences within this highly structurally conserved family of proteins allow for the differential regulation and cellular roles of eukaryotic AQPs.

## 2. Interactions between Aquaporin (AQP) Tetramers in Supramolecular Assemblies

From the various identified AQP classes and isoforms, only two AQPs have been shown to accumulate in supramolecular assemblies: mammalian AQP0 found in the eye lens [13], and AQP4, the main AQP in the brain [14,15]. These assemblies involve interactions within the same cell membrane (arrays) and between cells (junctions). AQP0 and AQP4 both form square-formed, so-called orthogonal arrays in their native membranes and AQP0 also mediates cell adhesion through its participation in thin junctions between lens fiber cells [13,14,15]. In both cases, the existence of shorter and longer AQP variants, seem controls the assembly process [13,16]. AQP4 has also been suggested to be involved in cell adhesion [17], however this is a controversial issue and remains to be conclusively shown.

### 2.1. AQP0

AQP0, originally named Major Intrinsic polypeptide (MIP), is exclusively found in the eye lens where it is the most abundant protein in the lens fiber cells, accounting for approximately 50% of the membrane protein content [18]. It is present in non-junctional membranes where it functions as a water pore [19], albeit at low efficiency compared to other water-conducting AQPs [15,19,20,21,22,23,24]. AQP0 is particularly enriched in the 11–13 nm thin junctions connecting fiber cells in the lens core where it forms interactions between AQP0 orthogonal arrays in the fiber cell membrane, thereby mediating cell-to-cell contact. Structural studies suggest that in its junctional form, the AQP0 pore is closed and does not conduct water, indicating that, unlike connexin channels in the thicker 16–17 nm gap junctions, AQP0 does not mediate transport between cells [25]. AQP0 thus has a dual role in the eye lens, facilitating transmembrane water transport to maintain the lens microcirculation system [26,27] and upholding the compact structure that is characteristic for the lens core through its cell adhesion properties. Both roles are important for preserving lens transparency and mutations in AQP0 are known to cause human cataracts [28,29,30,31,32].

#### 2.1.1. AQP0 Junction Formation Is Regulated by Proteolytic Cleavage

Studies have shown that AQP0 junction formation is controlled by proteolytic cleavage. This is an age-dependent process, with AQP0 in young fiber cells within the lens cortex, existing mainly in its full-length non-junctional form. As the fiber cells mature and grow older, they become more and more buried into the lens core. This correlates with a fraction of the AQP0 molecules becoming proteolytically cleaved at the N- and C-termini, an event that is believed to induce junction formation [13]. 2D crystallization of sheep AQP0 purified from the lens core, containing a mixture of full-length and truncated proteins yielded double-layered crystals with lattice dimensions (a = b = 65.5 Å) and thickness (11 nM) essentially the same as those found for AQP0 arrays in lens fiber junctions [33]. In contrast, 2D crystallization of full-length AQP0 purified from the lens cortex yielded single layer crystals, whereas reconstitution attempts using full-length chymotrypsin-treated AQP0 chymotrypsin produced large membrane stacks with an average distance of 5.5 nm, i.e., an overall thickness of 11 nm for two interacting membranes [13]. These results show that proteolytic cleavage of AQP0 increases its propensity for junction formation.

**Figure 3:**
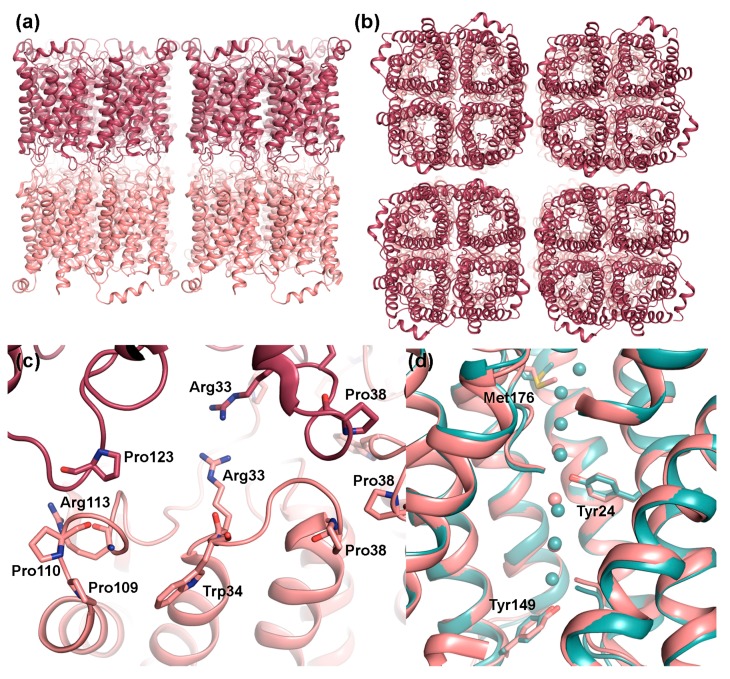
Structural features of AQP0 in junctions. Doubled layered 2D crystals of sheep AQP0 (PDB code 2B6O) viewed: (**a**) parallel and (**b**) perpendicular to the membrane plane. The tetramers in the two adjacent membranes are perfectly in register. (**c**) Close-up of residues involved in the interaction between tetramers. Within loop C, Pro109 and Pro110 interact with same motif in the opposite layer (not shown in figure) while Pro123 interacts with Arg113. Interactions also involve Arg33 and Pro38 in loop A. In non-junctional AQP0, the side-chains of Arg33 and Trp34 swap positions, blocking the interaction with the opposite tetramer, and Pro38 moves away from the tetramer centre. (**d**) Structural comparison of junctional (pink) and non-junctional (turquoise, PDB code 1YMG) AQP0 showing how the side-chains of Met176, Tyr24 and Tyr149 constrict the water pore. Pore closure upon junction formation is believed to be mediated by the difference in side-chain conformation of Met176.

Electron crystallographic analysis of junctional AQP0 2D-crystals revealed that within the junctions, AQP0 tetramers align on top of each other with residues in the extracellular loops A and C interacting with the corresponding residues in the adjacent layer [34,35] (Figure 3a,b). Specifically, these residues include a Pro-Pro motif (Pro109 and Pro110), Arg113 and Pro123 within loop C. In loop A, junctional contacts are mediated by Pro38, forming a rosette-like structure at the center of the tetramer, as well as by Arg33 and Trp34 (Figure 3c). Comparison with the X-ray structure of bovine AQP0 in its non-junctional form shows that, while the structure of loop C remains the same, a rearrangement of loop A cause Trp34 (sheep AQP0 numbering for comparison) to create steric hindrance for the interacting tetramer and Pro38 to move away from tetramer center [36]. Interestingly mutation of Arg33 to cysteine, causes congenital cataract in humans [37] and has impaired cell adhesion properties in vitro [32], supporting of role of loop A and this residue in AQP0-mediated cell adhesion. However, the mechanism behind how proteolytic cleavage of the N- and C-termini on the cytoplasmic side serves as a conformational switch to induce these structural changes on the extracellular side remains to be shown.

#### 2.1.2. Junctional AQP0 Is Closed

Structural studies of non-junctional [36] and junctional [34,35] AQP0 reveal an unusually narrow water-conducting channel. In the middle of the channel, Tyr24, a residue not found in other mammalian AQPs, points into the channel, breaking the hydrogen-bonding pattern of the single-filed water molecules (Figure 3d). This may be the reason why AQP0 conducts water at a slower rate than other water-specific AQPs, such as AQP1 and AQP4, where an uninterrupted chain of channel water molecules is observed [38,39]. In non-junctional AQP0, the presence of water molecules within hydrogen-bonding distance on either side of Tyr24 supports its ability to conduct water. In junctional AQP0, however, only three water molecules were found with distances too far apart to form hydrogen-bonding contacts. Close examination of the channel dimensions revealed, that the selectivity filter, the narrowest point of the channel and normally defined by the conserved aromatic/arginine region (ar/R), is extended towards the extracellular side due to a side-chain rotation of Met176 (Figure 3d). Furthermore, an additional constriction region is observed towards the cytoplasmic side, where Tyr149 points into the channel. These constrictions restrict water access from both the cytoplasmic and extracellular side, suggesting that in its junctional form, AQP0 is in a closed conformation. Since proteolytic cleavage does not affect AQP0 water permeability [40], it is likely that it is the junction formation itself that leads to channel closure, converting AQP0 from a water channel to a pure adhesion molecule. The most prominent difference between the open and closed pore of AQP0 lies in the side chain position of Met176, but exactly how junction formation triggers this conformational change is not known.

### 2.2. AQP4

AQP4 is expressed in several tissues throughout the body, but has received the most attention for being the main water channel in the brain and central nervous system [41,42,43]. It is found in brain glial cells and plays important roles in brain water homeostasis, neuroexcitation, astrocyte migration and neuroinflammation [44,45,46,47,48]. Due to its role in brain water balance, AQP4 is believed to play an important role in development of brain edema, which is supported by increased survival rates of AQP4 knock-out mice following traumatic brain injury [45]. AQP4 is also the target of pathogenic auto-antibodies in the multiple-sclerosis-like disease Neuromyelitis optica (NMO), causing nerve demyelination and inflammation which leads to paralysis and blindness [49,50]. As such, AQP4 in the brain has become an interesting target for drug development [51].

In cell plasma membranes, AQP4 forms regular square-formed arrays known as orthogonal array particles (OAP) [14,15]. These are particularly prominent in astrocyte end-feet, surrounding vascular capillaries in the brain [14]. The function of these OAPs is not completely known, but speculated roles include increased water permeability [52,53], AQP4 polarization to astrocyte end-feet [54] and cell adhesion [17]. OAP-formation is also of central importance in NMO pathogenesis, with NMO-Immunoglobulin G (IgG) antibodies having higher affinity towards AQP4 in OAPs than as individual tetramers and OAP-dependent clustering of NMO-IgG antibodies leading to enhanced complement-dependent cytotoxicity [55,56].

#### 2.2.1. AQP4 Orthogonal Array Formation Is Isoform-Dependent

In humans, alternative splicing results in two AQP4 variants, the longer AQP4-M1 (34 kDa) with translation starting at Met1 and the shorter AQP4-M23 (31 kDa) with translation initiation at Met23 [43,57]. Of these, only the shorter AQP4-M23 has been shown to form OAPs on its own, while AQP4-M1 exists mainly as individual tetramers [58]. However, when co-expressed, AQP4-M1 can assemble in OAPs together with AQP4-M23 as hetero-tetramers, reducing OAP size and affecting its shape [16,59,60]. Super-resolution imaging showed that OAPs have an M23-enriched core surrounded by an M1-enriched periphery [60]. It thus seems plausible that interactions between AQP4-M23 molecules provides the adhesive interactions that stabilizes the OAPs while the inability of AQP4-M1 to participate in these interactions limits OAP growth.

**Figure 4:**
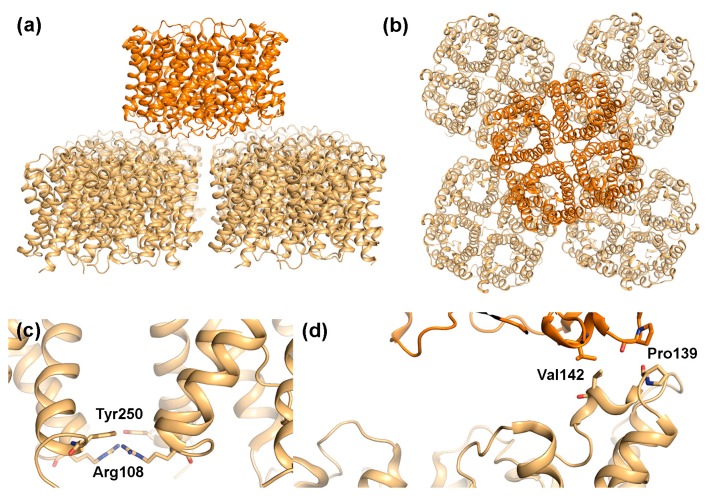
Structural features of AQP4 array formation. Double layered 2D-crystals formed by rat M23-AQP4 (PDB code 2D57) viewed: (**a**) parallel and (**b**) perpendicular to the membrane plane. One AQP4 tetramer interacts with four other tetramers in the adjacent layer; (**c**) Close-up of Arg108 and Tyr250, which mediate contract between tetramers on the extracellular side within membrane arrays and (**d**) Close-up of residues involved in interactions between the two layers in the crystal showing the involvement of Pro139 and Val142.

Structural studies of rat AQP4 suggest a mechanism by which AQP4-M23, but not AQP4-M1 is able to form OAPs. Electron diffraction analysis of AQP4-M23 2D-crystals shows tetramers arranged in a parallel fashion, interacting with each other via residues at the extracellular as well as the cytoplasmic side (Figure 4a,b) [17,61]. While interacting residues on the extracellular side are conserved in all AQPs, Arg108 and Tyr250, which form the two interaction sites on the cytoplasmic side, are specific for AQP4 (Figure 4c). It was hypothesized that in the AQP4-M1 isoform, Tyr250 instead interacts with the conserved Arg9 within the N-terminus, thereby preventing the interactions between tetramers on the cytoplasmic side that was observed in the 2D-crystals. However, single-molecule fluorescent imaging of AQP4 in live cells did not display any difference in OAP-formation when mutating Tyr250 to alanine [62]. Instead the ability of AQP4-M23 to form OAPs was attributed to hydrophobic interactions between residues just downstream of Met23 (Val24, Ala25, Phe26), while in AQP4-M1, residues just upstream of Met23 block these interactions in a non-specific manner. In addition, S-palmitoylation of two cysteine residues in the AQP4-M1 N-terminus play an important role in the ability of this isoform to disrupt OAP formation [59,63]. Unfortunately, structural studies by electron microscopy [17,61] as well as X-ray crystallography [38] have both failed to provide structural information for the AQP4 N-terminus beyond residue 31, due to disorder and truncation respectively. Therefore, the structural mechanism behind how AQP4-M23 form OAPs while AQP4-M1 prevents this remains to be conclusively shown.

#### 2.2.2. Does AQP4 Form Junctions?

**Figure 5:**
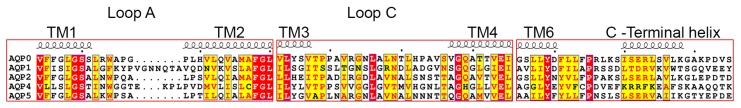
Sequence alignment of water-specific human AQPs. Sequences for human AQP0 (UniProt ID P30301), AQP1 (UniProt ID P29972), AQP2 (UniProt ID P41181), AQP4 (UniProt ID P55087) and AQP5 (UniProt ID P55064) were retrieved from the National Center for Biotechnology Information (NCBI) website (https://ncbi.nlm.nih.gov). Secondary structure information was taken from the electron crystallographic structure of sheep AQP0 (PDB code 2B6O). Loops A and C mediate contacts between adjacent tetramers in double-layered 2D crystals of AQP0 and AQP4. The C-terminus forms a short helix that in AQP0, AQP1, AQP2 and AQP5 have a similar distribution of hydrophobic and hydrophilic residues. The sequences were aligned using the T-Coffee server with PSI-TM Coffee option [70] (http://tcoffee.crg.cat) and the alignment was visualized using ESPript 3.0 [71]. Strictly conserved residues are highlighted in red and residues with a similarity score >0.7 are highlighted in yellow.

From the observed packing in the AQP4-M23 2D crystals, it was proposed that AQP4 may participate in junction formation [17,61]. Similar to AQP0, AQP4-M23 formed double-layered crystals with AQP4 tetramers interacting with each other via their extracellular regions (Figure 4a,b). However, unlike AQP0, where the interacting tetramers align perfectly on top of each other, the AQP4 layers are shifted, with one tetramer interacting with four other tetramers in the adjoining membrane, partially blocking the water-conducting channel. This suggests that junction formation is unlikely to occur in the absence of AQP4 orthogonal arrays. The interaction between opposing tetramers is mediated by two residues, Pro139 and Val142, situated in a short 3_10_-helix within extracellular loop C. Interestingly, Pro139 is part of the equivalent Pro-Pro motif seen to mediate junction contacts in AQP0 (Figure 3c). The Pro-Pro motif is not conserved in other mammalian AQPs (Figure 5), and thus may represent a unique junction-forming motif. In contrast to AQP0, variation in the relative position of the two AQP4 tetramer layers as well as the observation of partial layer separation suggest weaker adhesion properties of AQP4 arrays. This could indicate that when compared to AQP0, AQP4-mediated junctions are more dynamic. It has been suggested that rapid water flow through AQP4 could drive the interacting tetramers apart, resolving the junction [64]. If so, AQP4 junction formation may allow regulation of membrane water permeability in an AQP4-variant specific manner, with the stronger adhesive properties of larger OAPs being able to withstand larger osmotic differences.

While the structural analysis above indicates that AQP4-tetramers may form junctions, the cell adhesive property of AQP4 in vivo remains a controversial issue. An extensive study by Zhang and Verkman where several different cell lines were used, including primary glial cells and L-cells, the latter which lacks any endogenous adhesion molecules, failed to show a role of AQP4 in cell adhesion [65]. Furthermore, a peptide corresponding to the residues that was proposed to mediate the interaction between tetramers was not able to bind to the AQP4 extracellular surface. Nevertheless, it is possible that under some conditions, interactions between AQP4 tetramers in vivo does occur, as seen in a different study of AQP4-expressing L-cells where increased adhesive properties was detected [17]. Junctional membrane areas have been observed in the glial lamellae of the hypothalamus, and gold labeling indicates the presence of AQP4 in these junctions [17,66]. However, whether AQP4 is responsible for mediating junction formation in the glial lameallae or not remains to be determined

## 3. AQP Hetero-Tetramerization

While the majority of AQPs assemble as homo-tetramers, some AQPs have been observed to form hetero-tetramers consisting of different AQP variants. For mammalian AQPs, the hetero-tetramerization of AQP4-M1 and AQP4-M23 variants constitutes the most striking example, controlling formation of AQP4 OAPs as described in Section 2.2.1. Other examples include AQP2, in which case a mutant form of AQP2 causing autosomal-dominant nephrogenic diabetes insipidus (NDI), was able to form a hetero-tetramer with wild-type AQP2 [67]. As a result, AQP2 failed to target to the apical membrane of the collecting duct, causing the urine concentration defect that is the hallmark of NDI. However, the existence of “true” hetero-tetramers, consisting of different AQP isoforms, rather than AQP splicing variants or mutants assembling with the wild-type form, has only been reported for plant AQPs. Plant AQP hetero-tetramerization was first identified in lentil seed where two tonoplast-located AQPs (Tonoplast Intrinsic Protein, TIP) differing in size as well as in sequence were found to form a mixed tetramer [68]. The best-characterized example however is the hetero-tetramerization of AQPs found in the plant plasma membrane, (Plasma membrane Intrinsic Proteins, PIPs) for which hetero-tetramerization of the PIP1 and PIP2 isoforms has been suggested to be involved in regulating membrane permeability [69].

### 3.1. Plasma Membrane Intrinsic Protein (PIP) Hetero-Tetramerization Controls PIP1 Trafficking

PIPs are responsible for controlling the water permeability of the plant plasma membrane. They constitute a highly conserved family of proteins that traditionally have been divided into two phylogenetic groups: the PIP1 and PIP2, with later analysis suggesting further division of PIP2 into two clusters. PIP1 and PIP2 are highly homologous (~80%), with the main difference being the length of the N- and C-termini and extracellular loop A [69]. Nevertheless, they behave drastically different when it comes to function. While PIP2 has been well described as a water channel, most functional studies of PIP1 initially failed to reveal any solute conducting capability [72,73,74,75,76,77,78,79]. However, it was later shown that many PIP1 do not reach the plasma membrane on its own but that PIP1 trafficking to the plasma membrane depends on its interaction with PIP2 [80,81]. The observation that most PIP1 members are retained in the endoplasmatic reticulum (ER) and must interact with PIP2 to reach the plasma membrane has been described for several PIP1-PIP2 pairs from different plants and in different expression systems, and water conductance through PIP1 is now well established [81,82,83,84,85,86,87,88]. In addition to water, PIP1 members have been shown to be permeable to glycerol [72,77], boric acid [89] and CO_2_ [90]. While specific ER-export motifs have been described for PIP2 [91,92,93], these are lacking in PIP1, which instead is suggested to contain specific ER retention signals [91,93]. It is believed that the interaction with PIP2 hides the ER retention signal in PIP1 while the PIP2 export signal remains exposed [69].

Although the earliest results from PIP co-expression analysis are compatible with interactions between as well as within tetramers, recent studies have provided strong experimental evidence that PIP1 and PIP2 are able to form hetero-tetramers [94,95,96]. The prevalence of PIP1-PIP2 hetero-tetramers vs. PIP2 homo-tetramers is likely controlled by synchronized expression in time as well as location. This is supported by transcriptional analysis that have shown that PIP1 and PIP2 members that interact also correlate in expression levels during plant development as well as in response to different stimuli [97]. Due to the high sequence similarity, it could be argued that PIPs simply hetero-tetramerize as if they were identical molecules. However, this does not explain the behavior of all PIP1-PIP2 pairs, suggesting that there are specific mechanisms governing why certain PIPs form mixed tetramers.

### 3.2. PIP Hetero-Tetramerization and Substrate Conductance

Interestingly, hetero-tetramerization does not only allow PIP1 to reach the plasma membrane but also affects water conductance through the mixed tetramer. In oocytes, co-expression of PIP1 and PIP2 produces a larger increase in membrane water permeability than expression of PIP2 on its own [80] A study of the hetero-tetramerization of PIP1;1 and PIP2;1 from strawberry (*Fragraria x anassa*) showed increased water conductance through the PIP1-PIP2 hetero-tetramer compared with PIP1 and PIP2 alone [96]. In addition, hetero-tetramerization affected the pH-sensitive gating, which triggers channel closure at low pH through a conformational change of cytoplasmic loop D due to protonation of a conserved histidine [98,99]. For the hetero-tetramer, the pH sensitivity shifted towards more alkaline pH when compared to the FaPIP2;1 homo-tetramer. Thus, hetero-tetramerization with PIP1 would increase the probability of channel closure at more physiological pH. PIP hetero-tetramerization has also been suggested to influence the substrate specificity of the complex, in particular regarding CO_2_- and ion conductance [7,87]. Taken together, the combination of PIP1 targeting to the plasma membrane, modulation of water permeability and pH-sensitivity and the possibility of broadening substrate specificity points at PIP hetero-tetramerization is indicated as being a key regulatory mechanism for adjusting plasma membrane permeability in plants.

### 3.3. Structural Aspects of PIP Hetero-Tetramerization

To fully comprehend the molecular details of how PIPs form hetero-tetramers, high-resolution structural data of a PIP1-PIP2 complex will be crucial. In the absence of such information, site-directed mutagenesis, together with structural analysis and molecular modeling, has provided some clues regarding the structural aspects of PIP hetero-tetramerization.

#### 3.3.1. Loop A

**Figure 6:**
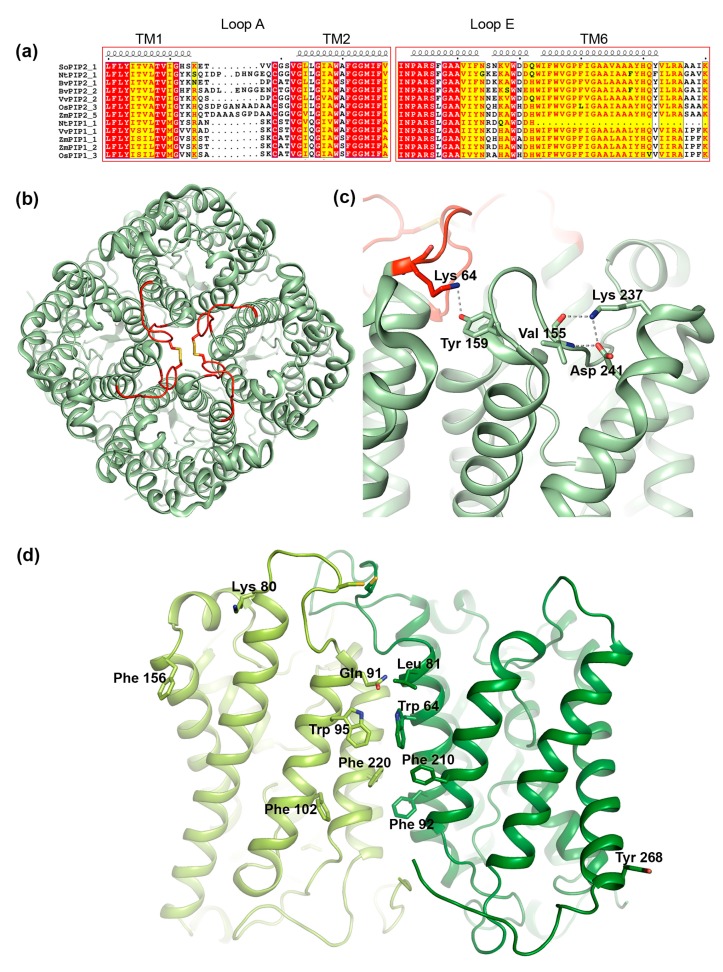
Structural features of PIP tetramerization. (**a**) Sequence alignment of selected members or the PIP1 and PIP2 subfamilies. The alignments includes the following sequences retrieved from the NCBI website (https://ncbi.nlm.nih.gov): *Spinacia oleracea* PIP2;1 (SoPIP2;1, UniProt ID Q41372), *Nicotiana tabacum* PIP2;1 (NtPIP2;1, UniProt ID Q8W506) and PIP1;1 (NtPIP1;1, UniProt ID Q8W507), *Beta vulgaris* PIP 2;1 (BvPIP2;1, GenBank accession code AAB67869.1) and PIP2;2 (BvPIP2;2, GenBank accession code ACT22630.1), *Vitis vinifera* PIP2;1 (VvPIP2;2, NCBI Reference sequence NP_001267957.1) and PIP1;1 (VvPIP1;1, NCBI Reference sequence NP_001267918.1), *Oryza sativa* PIP2;3 (OsPIP2;3, UniProt ID Q7XUA6) and PIP1;3 (OsPIP1;3, UniProt ID Q9SXF8), *Zea mays* PIP2;5, (ZmPIP2;5, UniProt ID Q9XF58) PIP1;1 (ZmPIP1;1 UniProt ID Q41870) and PIP1;2 (ZmPIP1;2, UniProt ID Q9XF59). Secondary structure information is taken from the crystal structure of SoPIP2;1 (PDB code 1Z98). Loop A, which mediates contacts between monomers in the centre of the tetramer contains a conserved cysteine but is otherwise variable in sequence and length. Loop E frequently contains residues of opposite charge that may be important for tetramer formation. The sequences were aligned using the T-Coffee server with PSI-TM Coffee option [70] (http://tcoffee.crg.cat) and the alignment was visualized using ESPript 3.0 [71]. Strictly conserved residues are highlighted in red and residues with a similarity score > 0.7 are highlighted in yellow. (**b**) Crystal structure of the SoPIP2;1 tetramer (PDB code 4JC6) viewed from the extracellular side of the membrane. Loop A is shown in red and the disulphide bond involving the fully conserved cysteine is shown in stick representation. (**c**) In SoPIP2;1, interactions are observed between Lys64 and Tyr159 in neighboring monomers as well as Lys237, Val155 and Asp 241 within the same monomer that may be important for tertiary and quaternary structure. (**d**) Homology model of a hetero-tetramer formed by ZmPIP2;5 (dark green) and ZmPIP1;2 (light green) [94]. Only two monomers are shown for clarity. Residues involved in hetero-tetramer formation are shown in stick representation.

One of the regions that have been suggested to play a role in interactions between PIP1 and PIP2 is the extracellular loop A. This is one of the most variable regions within the PIP subfamily, differing in sequence as well as in length (Figure 6a). Nevertheless, loop A contains a fully conserved cysteine that mediates a disulfide bond between PIP dimers, as seen in the crystal structure of PIP2;1 from spinach (Figure 6b) [88,90]. Bienert et al. showed that mutation of this cysteine does not change the ability of *Zea mays* PIP1;2 and PIP2;5 to interact, neither does it affect its trafficking nor its water permeability [76]. However, the mutation did alter its response to mercury, a common AQP inhibitor, rendering ZmPIP2;5 insensitive to mercury when co-expressed with the ZmPIP1 cysteine-to-serine mutant. This suggests that loop A somehow influences the conformation of ZmPIP2;5 within hetero-tetrameric arrangement. From a study of three PIPs from *Beta vulgaris* it was proposed that the reason BvPIP1;1 could interact with BvPIP2;2 but not BvPIP2;1 was due the PIP2 isoforms differing in two residues in the hinge region between transmembrane™ helix 1 and loop A (Figure 6a,b) [91]. The ends of loops have been shown to be particularly important for loop configurations [92], wherefore it was argued that these two residues might affect the dynamic behavior of loop A. We note that in the crystal structure of PIP2;1 from *Spinacia oleracea* (SoPIP2;1), the first of these two residues, Lys64, forms a hydrogen bond with a fully conserved tyrosine in loop C in the neighboring monomer, supporting the importance of this hinge region in tetramer formation (Figure 6c) [88,90].

#### 3.3.2. Loop E

Early studies of AQPs suggested that loop E which forms a half membrane-spanning helix on the extracellular side (Figure 1), is not only important for the water conductance mechanism but also for tetramer formation [100,101]. For PIPs, it was demonstrated that replacing loop E of ZmPIP1;1 with that of ZmPIP1;2 resulted in a increase in membrane permeability when co-expressed with ZmPIP2;5 in oocytes, an effect that was not observed for wild-type ZmPIP1;1 [80]. Molecular dynamics simulations suggested that these mutations alter the structure of the loop E half-helix and TM helix 6, thereby affecting tetramerization [102]. Sequence analysis reveal that the region between the loop E half helix and TM helix 6, frequently contain residues of opposite charge that may interact with each other or other residues close-by, constraining the loop E structure with possible implications for tetramer formation. In SoPIP2;1, we identified interactions between one of these charged residues, Lys237, and Asp241 in TM helix 6 as well as a backbone carbonyl within loop C that likely play an important role in maintaining tertiary as well as quaternary structure (Figure 6c) [98,103].

#### 3.3.3. Transmembrane Region

In addition to interactions between loop regions, interactions between TM helices help maintain the integrity of the AQP tetramer. Specifically, TM helices 1 and 2 form a left-handed coiled-coil with TM helices 4 and 5 in the adjacent monomer (Figure 6b), stabilizing the structure primarily via van der Waals contacts [98,103]. A clue about which specific residues form the interacting surface came from computer modeling of a hetero-tetramer between ZmPIP1;2 and PIP2;5, built from homology models of the respective protein based on the crystal structure of SoPIP2;1 [94]. Analysis of the generated set of models allowed for the identification of five and six putative interacting residues in ZmPIP1;2 and ZmPIP2;5 respectively. These were primarily located in the centre of the tetramer (Leu81/Gln91, Trp85/Trp95, Phe92/Phe102 and Phe210/220 in ZmPIP2;5/ZmPIP1;2) with a few residues residing in the peripheral regions (Lys80 and Phe156 in ZmPIP1;2 and Tyr268 in ZmPIP2;5 (Figure 6d). Notably Lys80 in ZmPIP1;2 is located in the loop A hinge region that was shown to be important for PIP1-PIP2 interaction in *Beta vulgaris* (see Section 3.3.1) and corresponds to Lys64 in SoPIP2;1 where it mediates contact between adjacent monomers in the tetramer (Figure 6c).

Substituting the putative interacting residues for alanine or their corresponding residues in ZmPIP1;2/ZmPIP2;5 failed to show any difference in interaction when expressed in oocytes, suggesting that hetero-tetramerization still occurs. However, several mutants inactivated water conductance through ZmPIP2;5 (W85A, F92A and F210A) or activated water conductance through ZmPIP1;2 (Q91L and F220A). Interestingly, the activating F220A mutation, located in ZmPIP1;2 TM helix 5 also inactivated ZMPIP2;5 within the hetero-tetramer. These data imply that the identified residues are involved in cross-talk between PIPs in the hetero-tetramer but that multiple mutations are required to disrupt the hetero-tetrameric state.

## 4. Transient Interactions between AQPs and Regulatory Proteins

Higher eukaryotes have evolved to post-translationally regulate their AQPs by gating or trafficking, allowing for control of membrane water permeability in a tissue-dependent manner and/or in response to environmental stress or hormonal triggers [74]. With the exception of plant AQP gating, where channel closure is mediated by a conformational change of the cytoplasmic loop D [98], protein–protein interactions play crucial roles in both regulatory mechanisms, either by directly affecting the pore dimensions or by governing the AQP trafficking process. For mammalian AQPs, a number of interaction partners have been identified [104], including (but not limited to) calmodulin (AQP0, AQP6) [105,106], the cystic fibrosis transmembrane conductance regulator (CFTR) (AQP4, AQP9) [107,108], α-syntrophin (AQP4) [109], the sulfonylurea receptor 1-transient receptor potential melastatin 4 (SUR1-TRPM4) channel (AQP4) [110], prolactin inducible protein (PIP) (AQP5) [111], perilipin-1 (AQP7) [112], ezrin (AQP0, AQP2) [113,114], the heat shock proteins Hsc70 and Hsp70 (AQP2) [115,116] and NEDD4 family interacting protein (NDFIP) 1 and 2; adaptor proteins for the neural precursor cell expressed developmentally down-regulated protein 4 (NEDD4) ubiquitin ligase (AQP2) [117]. These examples highlight the complexity and variability of the protein–protein interactions that different AQPs are involved in. A majority of AQP interaction partners bind to the C-terminus, although other regions have been proposed to be involved in the interaction with some partners [104]. The latter includes the interaction between AQP6 and calmodulin, which is suggested to be mediated by the AQP6 N-terminus [105]. The AQP C-terminus frequently harbors post-translational modifications sites, in particular phosphorylation sites. It is believed that post-translational modifications of these sites modulate the interactions with the regulatory proteins, allowing for dynamic control of the regulatory process [12,106,118]. In this review, we will use AQP0 and AQP2 to describe how protein–protein interactions mediate mammalian AQP regulation by gating and trafficking respectively. Both AQPs represent the canonical example for the respective regulatory process and have been structurally characterized [34,35,36,119], allowing for protein–protein interaction motifs to be discussed from a structural point of view.

### 4.1. Gating of AQP0 by Calmodulin

**Figure 7:**
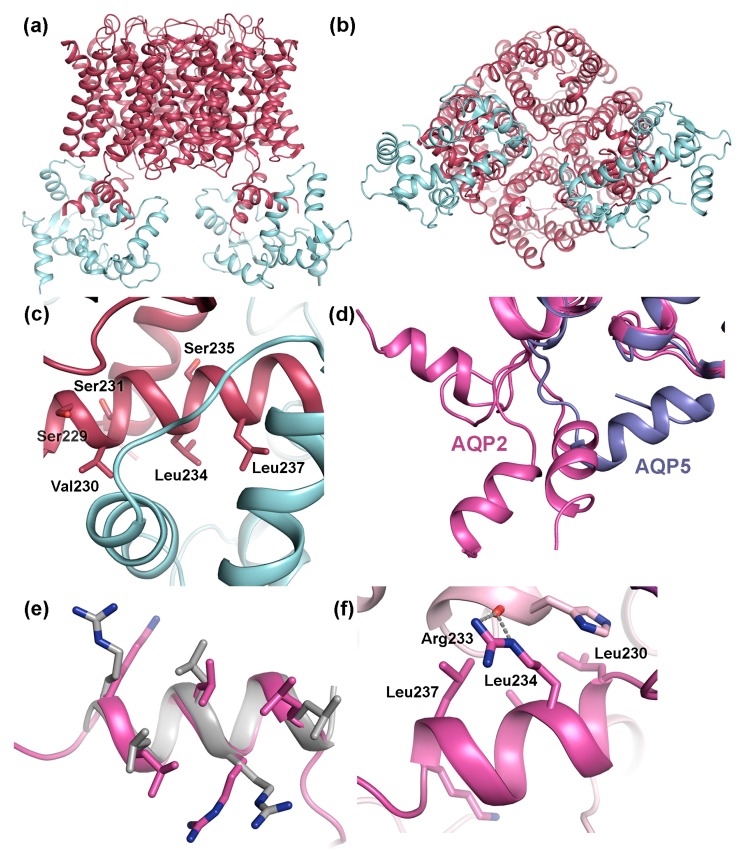
Transient interactions between mammalian AQPs and regulatory proteins. Pseudo-atomic model of the complex between AQP0 (dark red) and calmodulin (CaM, licht cyan) (PDB code 3J41) viewed: (**a**) parallel to the membrane; and (**b**) from the cytoplasmic side showing how one CaM-molecule binds the C-terminal helix from two AQP0 monomers. (**c**) Close-up of the AQP0-CaM interaction site showing three hydrophobic residues that are important for binding (Val230, Leu234, Leu237) as well as nearby phosphorylation sites (Ser229, Ser231, Ser235). (**d**) Crystal structure of human AQP2 (pink, PDB code 4NEF) showing how the C-terminal helix of the four overlaid AQP2 monomers adopts different conformations, neither of which correspond to the conformation seen in other mammalian AQPs, here illustrated by the crystal structure of human AQP5 (purple, PDB code 3D9S). (**e**) Overlay of the AQP2 C-terminal helix and the LYST-interacting protein 5 (LIP5) interaction site in charged multivesicular body protein 1B (CHMP1B) (grey, PDB code 4TXQ) showing how the MIT-interacting motifs (MIM) perfectly overlap structurally. (**f**) Close-up of AQP2 crystal contacts showing how the AQP2 MIM mediates interactions between symmetry-related molecules.

AQP0 is the only mammalian AQP for which regulation by gating has been conclusively shown. This involves binding of calmodulin (CaM), a ubiquitous Ca^2+^-binding protein that functions as a secondary messenger in several Ca^2+^-signaling pathways. In its Ca^2+^-bound form, CaM binds to to the C-terminal region of AQP0, thereby inhibiting water conductance in a Ca^2+^-dependent manner [80,84,106,120]. A pseudo-atomic model of the AQP0-CaM complex was obtained by fitting high-resolution AQP0 and CaM-structures into a 25 Å resolution single particle cryo-EM reconstruction [106]. In this model, one CaM-molecule binds two copies of a short helix within the AQP0 C-terminus, forming a 2:1 AQP0:CaM complex (Figure 7a,b). Studies of the interaction between a AQP0 C-terminal peptide and CaM indicated a step-wise binding process with a high-affinity event followed by a low-affinity event, and it was suggested that this corresponds to sequential binding of the two AQP0 C-terminal helices. Mutational studies revealed that CaM interacts with hydrophobic residues that cluster on one side of the C-terminal helix. (Figure 7c).

Phosphorylation of the AQP0 C-terminus abolishes Ca^2+^ dependent gating, presumably by weakening the interaction to CaM. Three phosphorylation sites are found within the CaM-binding region, Ser229, Ser231 and Ser235, all of which have been shown to be phosphorylated in the lens. [73] (Figure 7c). When expressed in oocytes, AQP0 mutants mimicking phosphorylation at Ser229 and Ser231 displayed increased water permeability that was insensitive to Ca^2+^, supporting that phosphorylation of these sites reduces CaM-binding. In the AQP0-CaM model, both Ser229 and Ser231 participate in hydrogen bonds with CaM. From a structural point of view, it therefore seems likely that phosphorylation would break these bonds, lowering the affinity of the AQP0-CaM complex.

#### Calmodulin Inhibits AQP0 Water Permeability through an Allosteric Mechanism

Analysis of the pore dimensions in the AQP0-CaM model showed that, although CaM binds directly below two of the AQP0 monomers in the tetramer, the channel remains open and is accessible for bulk water in the cytoplasmic vestibule [106]. This contradicts earlier suggestions that CaM acts as a plug, physically blocking the cytoplasmic channel entrance [80]. Molecular dynamics simulations indicated that, instead, CaM inhibits water conductance through an allosteric mechanism whereby CaM binding is communicated to a cytoplasmic constriction region near Tyr149 [106]. This constriction region, which has also been implied in channel closure upon junction formation (see Section 2.1.2 and Figure 3d), has been proposed as a dynamic channel gate, fluctuating between open and closed forms [34]. In its CaM-bound form, the movements of residues that form this gate were restricted compared to the CaM-free form, causing a significant shift in the equilibrium towards the closed conformation. Mutation of the conserved residues that forms the gate rendered AQP0 water permeability insensitive to Ca^2+^ when measured in oocytes, suggesting that CaM-dependent gating had been lost. It was therefore concluded that the interaction between AQP0 and CaM allosterically controls channel closure by coupling CaM-binding the conformational state of the cytoplasmic constriction region [106].

### 4.2. Trafficking of AQP2

The most common regulatory mechanism for mammalian AQPs is trafficking, whereby shuttling of AQP molecules between intracellular storage sites and the plasma membrane controls AQP membrane abundance and, consequently, membrane water permeability. A majority of the thirteen AQPs that have been identified mammals are regulated by trafficking in response to hormonal or environmental triggers, including AQP1, AQP2, AQP4 and AQP5 [121]. Of these, vasopressin-mediated trafficking of AQP2 in the kidney collecting duct is by far best characterized, a process that is crucial for our ability to regulate urine volume [122]. Upon dehydration, binding of the pituitary hormone vasopressin to the vasopressin receptor (V2R) in the basolateral membrane of the collecting duct principal cells triggers a cAMP-signaling cascade. This stimulates protein kinase A (PKA)-mediated phosphorylation of AQP2 residing in storage vesicles, triggering its translocation to the apical membrane [123]. Failure of AQP2 to reach the apical membrane leads to the water balance disorder Nephrogenic Diabetes Insipidus (NDI), characterized by excessive urine volumes and dehydration [122]. Multiple post-translational modification sites in the AQP2 C-terminus controls the trafficking process by acting as sorting signals for the cellular trafficking machinery. In particular, vasopressin-stimulated phosphorylation of Ser256 is of vital importance and has been shown to be crucial and sufficient for AQP2 translocation to the apical membrane [123]. Vasopressin also triggers additional phosphorylation at Thr269 (Ser269 in mouse), resulting in prolonged AQP2 apical membrane residence time as well as phosphorylation of Ser264, the function of which is currently not completely understood [124,125]. In contrast, Ser261 is dephosphorylated in response to vasopressin and AQP2 phosphorylated and this residue mainly resides in intracellular storage vesicles [126,127]. AQP2 removal from the apical membrane is stimulated by ubiquitination of Lys270, after which AQP2 may be stored again in vesicles or targeted to multi-vesicular bodies (MVB) for subsequent lysosomal degradation [128].

#### 4.2.1. Protein-Protein Interactions Control AQP2 Trafficking

During trafficking, membrane proteins are selectively recruited into transport vesicles that bud off one membrane-bound compartment, slides along the cytoskeleton and fuse with the next [129]. This is a highly coordinated process that involves interactions with proteins within the cellular trafficking machinery and is governed by membrane protein intrinsic sorting signals, for example post-translational modification sites, as described for AQP2 above, or short linear sequences [130]. For AQP2, a number of interacting proteins that effect its trafficking have been identified, most often binding to the AQP2 C-terminus. These interacting proteins include members of the general trafficking machinery such as clathrin heavy chain [115], Heat shock cognate protein 70 (Hsc70) [116,131], Annexin II [131,132] and LYST-interacting protein 5 (LIP5) [133] as well as cytoskeletal or cytoskeleton associated proteins such as actin, tropomyosin 5b and ezrin [113,134]. Several of the identified interaction partners bind AQP2 in a phosphorylation-dependent manner and it is believed that these altered affinities forms the basis for how post-translational modifications govern AQP2 trafficking.

The crystal structure of human AQP2 revealed an unusual C-terminal flexibility that had not been observed for other mammalian AQPs [119]. In particular, the cytoplasmic C-terminal helix, which is a shared structural feature between mammalian AQPs, adopts a different position in each AQP2 monomer relative to tetramer (Figure 7d). This is not seen in any other mammalian AQP-structure where the C-terminal helix always remains in a conserved position across the cytoplasmic interface [34,35,36,39,135]. The unique flexibility of the AQP2 C-terminus was attributed to a di-proline motif immediately prior to the C-terminal helix in AQP2 presumably acting as a hinge region, whereas only a single proline is present in the equivalent position in other mammalian AQPs. The conformational flexibility of the AQP2 C-terminus is likely to increase its accessibility as a protein–protein interaction site and therefore be may be important for its ability to bind proteins during the trafficking process.

#### 4.2.2. AQP2 Phosphorylation Allosterically Controls its Interaction with LYST-interacting protein 5 (LIP5)

In terms of binding site and phosphorylation-mediated regulation of binding, the interaction between AQP2 and the lysosomal trafficking regulatory protein LYST-interacting protein 5 (LIP5) constitutes the best-characterized example. LIP5 is involved in multivesicular body (MVB) biogenesis where it coordinates the actions of the endosomal sorting complexes required for transport (ESCRT)-III complex and the vacuolar protein sorting-associated protein 4 (VPS4) ATPase, leading to the formation of MVB inner vesicles [136,137]. During trafficking, LIP5 also binds membrane protein cargo that is destined for MVB’s, one of which is AQP2 [133]. The interaction between AQP2 and LIP5 leads to increased AQP2 lysosomal degradation and thus plays an important role in reducing the amount of AQP2 in the apical membrane. The LIP5-binding site in AQP2 has been localized to the C-terminal helix (Figure 6d). This region contains the amino acid motif LSERLAVLK, perfectly matching the MIM1 (MIT-interacting motif 1) consensus sequence LXXRLXXLK/R [138]. MIM1-motifs are known to be mediate LIP5-binding, interacting with an MIT-domain found in N-terminal part of LIP5 [137,138]. MIM1-motifs typically form amphipathic helices (as seen in AQP2) where the hydrophobic residues are exposed on one side. A structural comparison between the C-terminal helix of AQP2 and the LIP5 binding site in charged multivesicular body protein 1B (CHMP1B), one of the components of the ESCRTIII-complex whose interaction with LIP5 has been structurally characterized [137] reveal that the MIM1-motifs overlap perfectly (Figure 7e). Furthermore, in the crystal structure of AQP2, the MIM1-motif was seen to mediate contacts between symmetry-related molecules in the crystal in a similar manner as can be expected for the AQP2-LIP5 complex, supporting its ability to participate in protein-protein interactions (Figure 7f).

Very recently, Roche et al. provided the first insights into how the interaction between AQP2 and LIP5 is regulated by AQP2 phosphorylation [76]. In this study, the affinity between AQP2 and LIP5 was determined for wild-type AQP2 as well as for a set of phospho-mimicking AQP2 mutants (S256E, S261E, S264E, T269E, and S256E/T269E). It was shown that phosphorylation of AQP2 significantly reduced LIP5 affinity in all cases but one (S264E), despite the fact that they lie outside the LIP5 binding region. This fits well with their respective role in AQP2 trafficking, since neither AQP2 phosphorylated at Ser256, Thr269 or Ser261 should be targeted to MVBs. Phosphorylation of Ser264 on the other hand has been proposed to be involved in exosome excretion of AQP2. Since exosomes originate from MVB inner vesicles [139], this could explain the retained affinity between AQP2 S264E and LIP5. Interestingly, removal of all phosphorylation sites by truncation resulted in the lowest LIP5 affinity, suggesting that the distal C-terminus that contains the phosphorylation sites is somehow involved in LIP5 binding. It was proposed that phosphorylation of AQP2 serves as an allosteric trigger, inducing structural changes to the C-terminus that affects its interaction with LIP5 or modulating as secondary binding site within the distal C-terminus.

### 4.3. The AQP C-Terminal Helix—A Common Motif for Protein-Protein Interactions?

**Figure 8:**
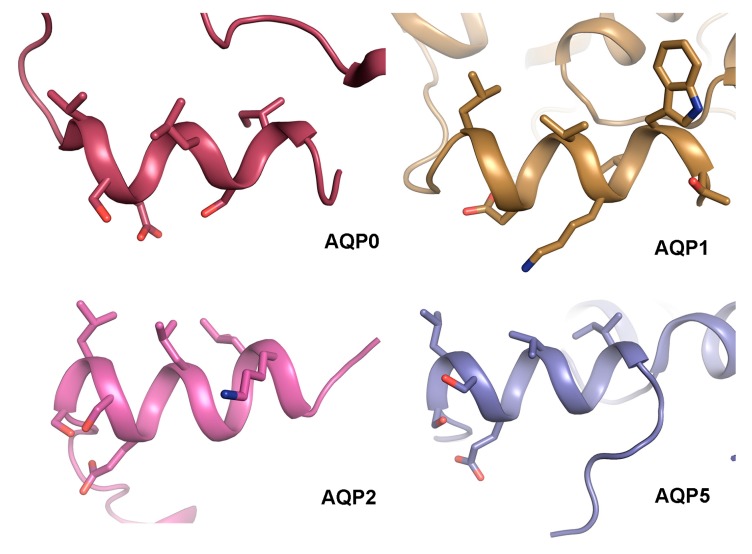
Structural comparison of the AQP C-terminal helix. Crystal structures of sheep AQP0 (PDB code (2B6O), bovine AQP1 (PDB code 1J4N), human AQP2 (PDB code 4NEF) and human AQP5 (PDB code 3D9S) reveal that their C-terminus forms an amphipathic C-terminal helix with hydrophobic residues lining up on one side. In AQP0 and AQP2, these residues mediate contact with calmodulin and LIP5 respectively.

Comparison of the structural basis for how AQP0 and AQP2 interact with calmodulin and LIP5 respectively reveals a striking similarity: both calmodulin and LIP5 interacts with analogous hydrophobic motifs within the AQP C-terminal helix. This makes it tempting to speculate that the C-terminal helix presents a common protein–protein interaction motif amongst human AQPs. Structural analysis of AQP0 [34,35,36], AQP1 [39], AQP2 [119] and AQP5 [135] shows that the C-terminal helix does indeed share an amphipathic character with hydrophobic residues lining up on one side (Figure 8). While protein–protein interactions involving this region are well established for AQP0 and AQP2 [76,106,133], this has not yet been shown for AQP1 and AQP5. Nevertheless, the C-terminal region of AQP5 is known to interact with Prolactin-inducible protein (PIP), thereby increasing AQP5 apical membrane abundance in salivary glands [111], although an involvement of the C-terminal helix remains to be shown. Furthermore, AQP5 trafficking is suggested to be controlled by PKA-phosphorylation at Ser156 and Thr259, the latter corresponding to Ser256 in AQP2 [140]. Since phosphorylation of Ser256 in AQP2 alters its affinity to LIP5, which is known to bind to the C-terminal helix, it may be that phosphorylation of AQP5 at the corresponding site controls trafficking by similar structural effects.

To our knowledge, there are no identified interacting partners that bind the C-terminus of AQP1. However, PKC-mediated phosphorylation of a threonine residue within the AQP1 C-terminal helix (Thr239) increases membrane water permeability in oocytes [78]. Since PKC is involved in regulating AQP1 trafficking, this is most likely due to an increase in AQP1 membrane abundance [79]. It thus seems plausible that AQP1 phosphorylation of Thr239 would alter its affinity for interacting proteins that control trafficking, although the identity of these proteins remains to be elucidated. This indirectly points at the C-terminal helix as a protein–protein interacting motif also in AQP1.

## 5. Conclusions

In the last two decades, structural studies of AQPs have made this one of the structurally best characterized membrane protein families, providing detailed understanding of the conduction mechanism, substrate specificity and regulation [12]. However, to fully understand AQP function, we must also take into account interactions with other proteins, without which tissue-dependent AQP regulation would not be possible. Due to the high structural similarities between AQPs, understanding these tissue-specific regulatory mechanisms is crucial to exploit them as drug targets for human disease. In this respect, taking the step from solving individual AQP structures to structurally characterizing AQP-interacting protein complexes at high spatial resolution will be a tremendous leap forward. We hope that this review will serve as inspiration for future research endeavors within this area.

## Abbreviations

AQP	Aquaporin
PIP	Plasma membrane intrinsic protein
CaM	Calmodulin
MIM	MIT interacting motif
MIT	Microtubule interaction and trafficking
MVB	Multivesicular body
LIP5	LYST-interacting protein 5
